# Advances and Applications of Water Phytoremediation: A Potential Biotechnological Approach for the Treatment of Heavy Metals from Contaminated Water

**DOI:** 10.3390/ijerph18105215

**Published:** 2021-05-14

**Authors:** Cristián Raziel Delgado-González, Alfredo Madariaga-Navarrete, José Miguel Fernández-Cortés, Margarita Islas-Pelcastre, Goldie Oza, Hafiz M. N. Iqbal, Ashutosh Sharma

**Affiliations:** 1 Área Académica de Ciencias Agrícolas y Forestales, Instituto de Ciencias Agropecuarias, Universidad Autónoma del Estado de Hidalgo, Tulancingo 43600, Mexico; cristian_delgado@uaeh.edu.mx (C.R.D.-G.); alfredo_madariaga@uaeh.edu.mx (A.M.-N.); mislas@uaeh.edu.mx (M.I.-P.); 2 Centre of Bioengineering, School of Engineering and Sciences, Tecnologico de Monterrey, San Pablo 76130, Mexico; A01208134@itesm.mx; 3 Centro de Investigación y Desarrollo Tecnológico en Electroquímica (CIDETEQ), Parque Tecnológico, Pedro Escobedo 76703, Mexico; goza@cideteq.mx; 4 School of Engineering and Sciences, Tecnologico de Monterrey, Monterrey 64849, Mexico

**Keywords:** hazardous pollutants, toxic elements, removal mechanisms, bioremediation, biodiversity, water

## Abstract

Potable and good-quality drinking water availability is a serious global concern, since several pollution sources significantly contribute to low water quality. Amongst these pollution sources, several are releasing an array of hazardous agents into various environmental and water matrices. Unfortunately, there are not very many ecologically friendly systems available to treat the contaminated environment exclusively. Consequently, heavy metal water contamination leads to many diseases in humans, such as cardiopulmonary diseases and cytotoxicity, among others. To solve this problem, there are a plethora of emerging technologies that play an important role in defining treatment strategies. Phytoremediation, the usage of plants to remove contaminants, is a technology that has been widely used to remediate pollution in soils, with particular reference to toxic elements. Thus, hydroponic systems coupled with bioremediation for the removal of water contaminants have shown great relevance. In this review, we addressed several studies that support the development of phytoremediation systems in water. We cover the importance of applied science and environmental engineering to generate sustainable strategies to improve water quality. In this context, the phytoremediation capabilities of different plant species and possible obstacles that phytoremediation systems may encounter are discussed with suitable examples by comparing different mechanistic processes. According to the presented data, there are a wide range of plant species with water phytoremediation potential that need to be studied from a multidisciplinary perspective to make water phytoremediation a viable method.

## 1. Introduction

One of the current challenges due to the global population is providing clean water to the whole world. According to the World Health Organization (WHO) [1], around 2.2 billion people use non-potable and untreated water services from highly unsafe water resources. Some examples of studies in water pollution [2,3] have registered the potential impact of water contamination by heavy metals (HMs), demonstrating a high risk for health issues, such as carcinogenicity and other diseases, associated with polluted water bodies. To keep providing safe and clean water to every person globally, maintenance and infrastructure need to be assured. However, underdeveloped countries often do not have access to services that ensure water quality for distribution and human consumption [4].

The Water Quality Index and other indexes help to understand factors that may influence water quality and make decisions on drinking water quality based on the criteria, such as pH, microorganisms in water, persistence of organochlorides, and heavy metals (HMs), among others [5,6], but not every country has access to water that meets the proposed criteria [7].

The sources of water contamination may be diverse. Nevertheless, they can be grouped into four broad categories: pathogens, inorganic compounds, organic material, and macroscopic pollutants. Inorganic and organic contaminants are the most common, even in treated water [8,9]. Furthermore, inorganic pollutants are more persistent in both waste and treated waters [10,11,12,13,14,15,16,17]. According to the WHO [1], HMs contaminate water, with mercury (Hg), cadmium (Cd), arsenic (As), chromium (Cr), and lead (Pb), among other heavy metals, having a long persistence in the environment. Heavy metal contamination is detrimental to health after prolonged consumption, being linked to conditions such as behavioral disorders, respiratory problems [18], oxidative stress cause by reactive oxygen species (ROS) [19], and immune, skin, respiratory, and endocrine diseases [20], among others. By acknowledging these statistics, we can recognize contaminated water as a global issue that needs to be addressed.

However, the variety of effects a pollutant can cause depends on the nature of the contaminant. HMs represent some of the most common and hazardous pollutants, widespread in soil and water. Some of them (such as Cu, Ni, Mn, Zn, and Co) play an important role in plant metabolism [21]. Many others are hazardous elements to both plants and animals, according to data presented by the United States Environmental Protection Agency [22]. HMs such as As, Hg, Cr, Cd, and Pb can cause hazardous effects in the cardiovascular, dermal, respiratory, and digestive systems, and many other pollutants cause diverse health effects, including asthma, diabetes, cancer, and Parkinson’s disease [23]. 

Electrochemical, chemical, and physical techniques are used to treat some contaminants, but may not reduce them. Soil leaching, adsorption, and nano-sorbents are some of the strategies that remove metals from soil [24]. This is why emerging technologies, such as bioremediation, can be implemented to improve remediation systems—for example, the usage of plants to remove some of the most common pollutants in water. In this review, we address the importance of water phytoremediation techniques for heavy metal (HM) pollutants, analyzing previous studies, specific plant species, biological processes, developed technologies based on patents and the current needs and research gaps.

## 2. Materials and Methods

A total of 435 articles were analyzed, but only 170 from 2000 to 2020 were discussed as a part of the phytoremediation systems review, following the year of publication, evidence of phytoremediation, properly described physiological process, and positive results on the removal of the contaminant. However, studies that may not include adequately described methods or supported results were excluded. We used PubMed, SCOPUS and other free data search tools to look for relevant articles, using the following keywords: water phytoremediation, phytoremediation mechanisms, water plants, and water pollutants, selecting 40 different species from 22 families for further analysis, according with the mobilization of HM results and the adequate explained methodology. Non-parametric estimators of species richness were used in Estimates (9.1.0) to determine an approximate number of species associated with phytoremediation processes in water to assure the representativeness of the data [25,26]. A search in Google Patents was conducted to compare the 40 selected species, with the total of patents associated with each species. Only water phytoremediation-related patents were considered for this comparison. 

## 3. Results and Discussion

Phytoremediation has been primarily used to treat soil pollutants [27,28,29,30]. With emerging hydroponic methods and a mechanistic approach [31], the usage of plants to bioremediate water has become more available. However, to establish a replicating water phytoremediation model, the morphological and physiological traits associated with the pollutant uptake, compartmentalization, volatilization, filtration, and many other processes must be understood. Many studies (Table 1) have shown the importance of plants for treating pollutants, both inorganic and organic, even those pollutants that might be recalcitrant or difficult to metabolize. HMs represent important pollutants that may remain in water and sediments [32], that different strategies try to treat [33]. However, phytoremediation represents an emerging technology that can help in HM removal [34].

**Figure 1:**
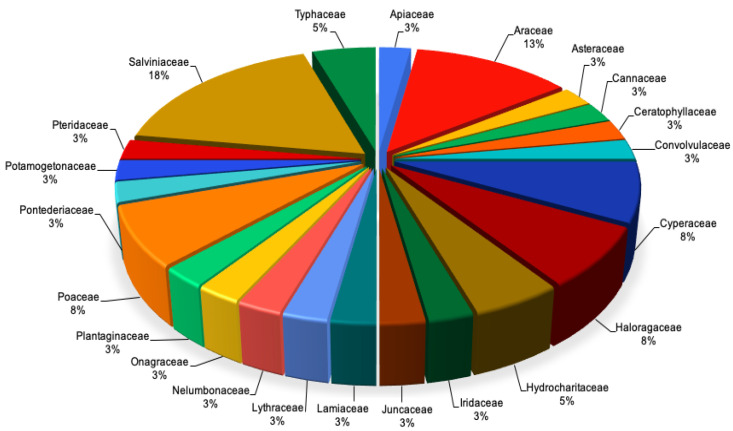
Twenty-two families corresponding to 40 species reviewed in the present study reported due their water phytoremediation capacity. The higher the percentage, the higher the representation in the analysis, implying more species for each family. References are listed in Table 1.

Biodiversity is a point that needs to be explored in water phytoremediation systems, since diversity may infer different metabolic pathways, resistance and mobilization of HM characteristics in plants [81,82,83]. Since there are several diseases associated with these pollutants, and each of them may vary in its bioavailability and chemical structure, different mechanisms and plants have to be used to remove these pollutants. The five most representative plant families (Table 1, Figure 1) were Salviniaceae, Araceae, Cyperaceae, Haloragaceae, and Poaceae, which together conform to almost 55% of the total plant species. This allows us to infer that knowledge of plants and their usage as phytoremediators in water needs to be extended, since the number of plant species reported is more than 374,000 in total and is increasing each year [84].

Salviniaceae, the most representative family, with almost 18% of the total species, is characterized by free-floating plants. Even though most of the species lack a developed root system [85], several studies reported their water phytoremediation capability. The two genera within the family are represented in the review, *Azolla* and *Salvinia* spp., but only 6 out of the 16 total reported species have been studied under phytoremediation criteria [85,86]. The second family with the most presented species is Araceae, with almost 13% of the 40 reported plants. However, in this family, there are 3800 published species with 102 genera, according to the most recent family-wide molecular phylogeny published in 2008 [87]. By comparing the results of Table 1, we can observe only five species out of the total 3800 reported species that have been studied, and only four genera from the 102 previously reported. The last three most representative families, with almost 8% of the total diversity, are Cyperaceae, Haloragaceae, and Poaceae. 

This means that the 24% of the total species are represented by the Magnoliophyta division, and comparing the total species of each family, less than 5% of the total reported species have been analyzed under phytoremediation criteria [88,89,90,91]. One of the data’s common factors is the lack of research related to phytoremediation, in contrast with the total number of registered species worldwide. Only a few species from the same genera or family have similar phytoremediation properties. This means that there is a high probability of their usefulness in water phytoremediation systems. From the data presented above, we can find that only 15% of the total species (6 out of the 40) are terrestrial plants, which means that their water requirements are lower than the other 85% of the plants from the table. However, the radicular system can be modified and adapted to water conditions [92,93], which may be one important factor to allow the filtration and mobilization of the contaminants from the water to plant tissue.

The present study considers research focused mainly on hydroponic controlled systems; even though constructed wetland studies have shown an important removal of HMs [94,95], the source of the water, mainly wastewater [95,96], and other non-controlled variables may affect the evaluation of the actual participation of the plant inside the system, such as interaction with other contaminants [97] and positive or negative interactions between different native plants species [98], among others. The comparison in the present review is focused on studies that can be compared likewise, with most of the variables under control, such as pH and temperature, among others. The physiology and mechanisms of mobilization for each plant may be similar, but many differences need to be addressed to understand the overall phytoremediation system in depth.

## 4. Mechanistic Approach

**Figure 2:**
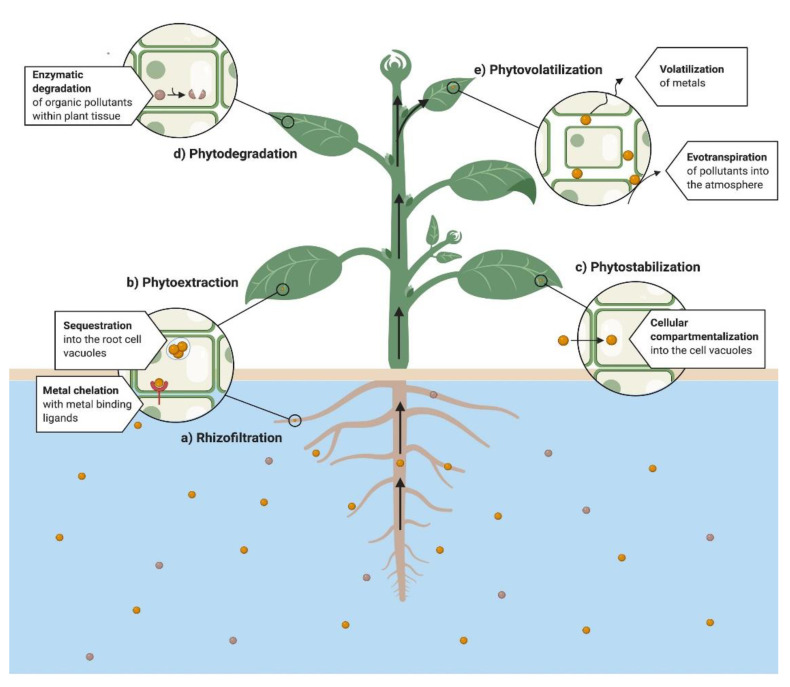
General diagram of phytoremediation processes in water.

Plants are viable for use in the remediation of the organic and inorganic pollutants. (Figure 2) [99]. Based on the process and application, phytoremediation has been commonly divided into the following mechanisms, i.e., rhizofiltration, phytovolatilization, phytodegradation, phytostabilization and phytoextraction [100,101,102,103,104,105,106,107]. The phytoremediation capacity of a plant depends on the molecular and physiological mechanisms [105]. In the same way, understanding the biological processes implicated in the phytoremediation technique is imperative to improve their efficiency [103], which implies significant tolerance to pollutants, greater accumulation capacity [99], higher plant yield and significant pollutant uptake [104]. We discuss below the main properties for each technique applied to phytoremediation mechanistically.

### 4.1. Phytoextraction

**Figure 3:**
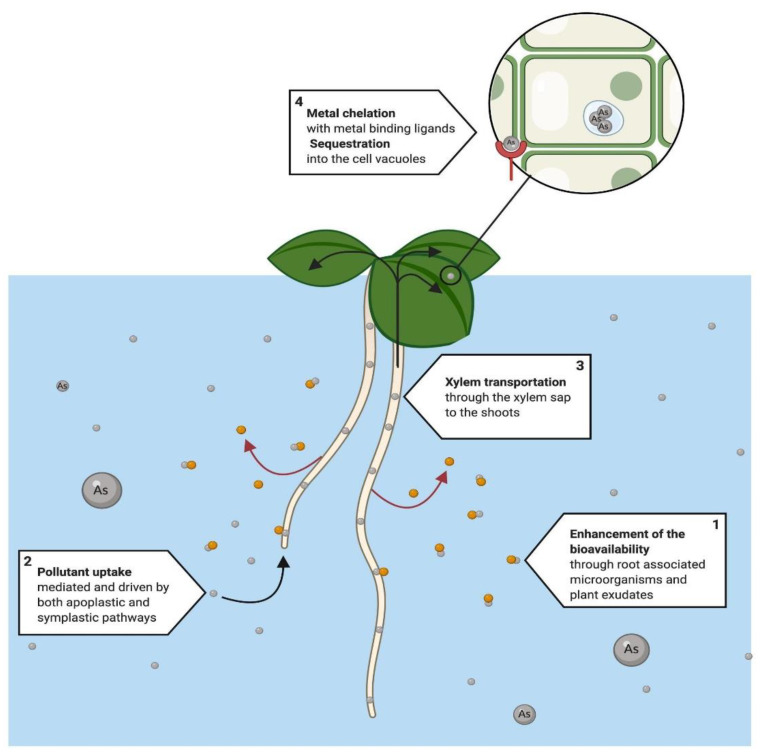
Proposed phytoextraction system that has been mainly used for the removal of heavy metals, such as arsenic (As). The process initiates with the root uptake of the metals (1). Prior to absorption, (2) the bioavailability of metals can be enhanced through root associated microorganisms and plant root metabolites, to improve in the phytoextraction process. Once the metal is available, (3) it is mobilized to shoots and leaves, through the xylem sap. Finally, (4) the pollutant is chelated and sequestered by the cell into the cell vacuoles where it will later will be harvested within the plant tissue for proper disposal [113,114,115].

The amount of HM that a plant can accumulate is determined by the capacity of the plant species to sequester the compound or metal and the intercellular mobilization through the plant [104]. Accumulation is a complex process that involves several steps, which include heavy metal transportation through the plasma membrane of root cells, xylem loading, translocation, sequestration, and detoxification at cellular levels in the whole plant [108]. During the absorption, pollutants’ bioavailability can be enhanced through root-associated microorganisms [109] and root exudates [110], and even in hydroponic cultures, microorganisms can establish a symbiotic relationship with the plant’s roots [111,112]. There are several reports about the potential of specific plants for metal phytoextraction in water that can be compared with the current knowledge of the physiological traits [35,75] (Figure 3).

### 4.2. Rhizofiltration

**Figure 4:**
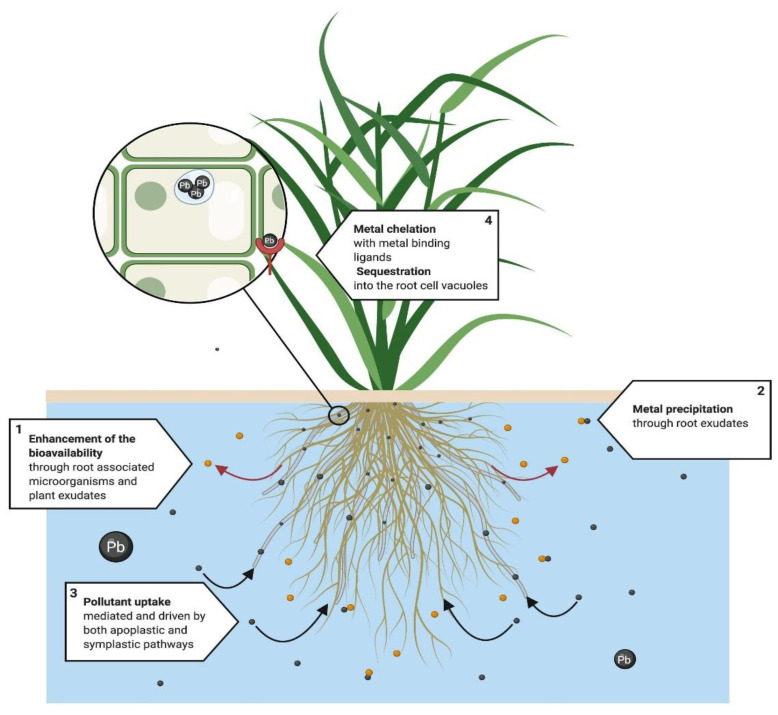
Proposed rhizofiltration system. This technique is mainly used in metal phytoremediation. From a physiological perspective, root exudates and microorganisms (1) enhance bioavailability, followed by (2) the precipitation of metals. (3) Pollutant uptake is mediated and driven by both apoplastic and symplastic pathways. If symplastic pathways fail to translocate the pollutant into leaves and steam, chelation and sequestration takes place mainly in the roots of the plant. (4) Metals are chelated by metal binding ligands, phytochelatins and metalloteines, and finally pollutants are either sequestered into the cell vacuoles and the apoplast or bound to the cell wall [114,115,116,117].

Rhizofiltration (Figure 4) occurs when plant roots remove pollutants, principally metals, from an aqueous substrate [116]. It involves the absorption, adsorption [99], and precipitation of pollutants from the substrate [116], which first take place in the root surface through different interrelated physicochemical processes, such as chelation, ion exchange, and chemical precipitation through root exudates, among others [116].

Metal uptake in the roots occurs under the same mechanisms as in the phytoextraction process. However, for the rhizofiltration process, the translocation in the plant cannot be achieved efficiently since the contaminant accumulates mainly in root tissue [114]. After its uptake and mobilization, the pollutant is stored in the vacuoles and the apoplast, or covalently bonded to the cell walls [117], after being chelated by phytochelatins and metallothioneins [115]. Evidence of metal rhizofiltration in water has been shown in several studies [64,78], with high concentrations in root tissue [63,66].

### 4.3. Phytodegradation

**Figure 5:**
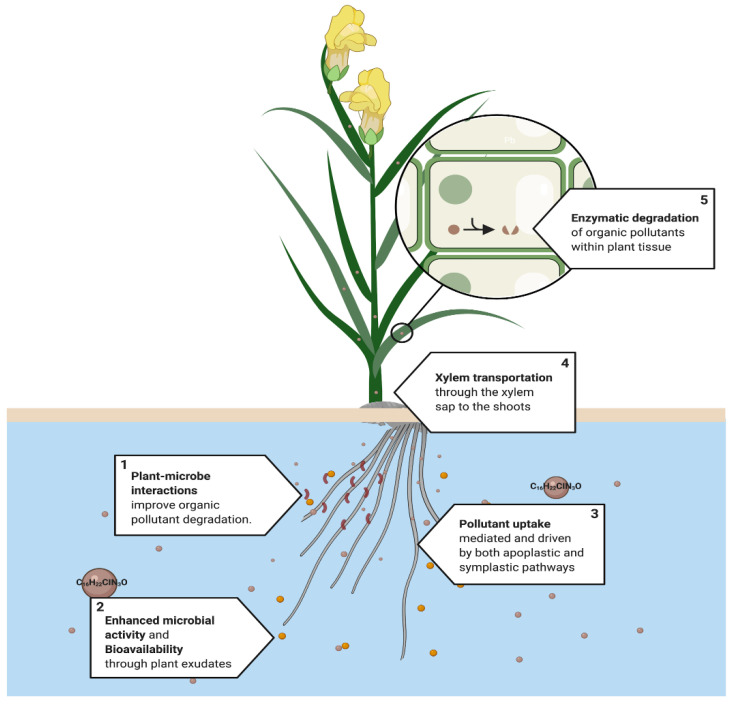
Proposed phytodegradation system. This process is used mainly in the phytoremediation of organic pollutants, such as tebuconazole C16H22CIN2O. (1) Plant–rhizosphere interactions improve the degradation of organic pollutants. (2) Root exudates increase the bioavailability and enhance rhizospheric activity, (3) followed by pollutant uptake, which occurs in the roots and is mediated by apoplastic and symplastic pathways. (4) Then, organic pollutants can be transported to mainly leaves or the roots through the xylem sap to finally (5) be metabolized in the cell to less toxic compounds by the action of plant enzymes. [111,112,120,121].

Pollutants can either be entirely mineralized into inorganic compounds or degraded to a stable, less toxic intermediate that is attracted by the cell wall or sequestered by the vacuoles [118]. Enzymes serve as biological catalysts for degradation within the plant tissue [119]. Rhizosphere pollutant degradation improves with an increased microbial metabolic activity and growth, which are enhanced by plant exudates [111,112,120,121]. Thus, plant–microbe interactions are an essential mechanism to achieve the degradation of organic pollutants [122,123,124]. Many examples of the participation of phytodegradation have been reported, which helps us to understand the physiological dynamics and the metabolic pathways that are associated with it [47,125] (Figure 5). 

### 4.4. Phytostabilization

**Figure 6:**
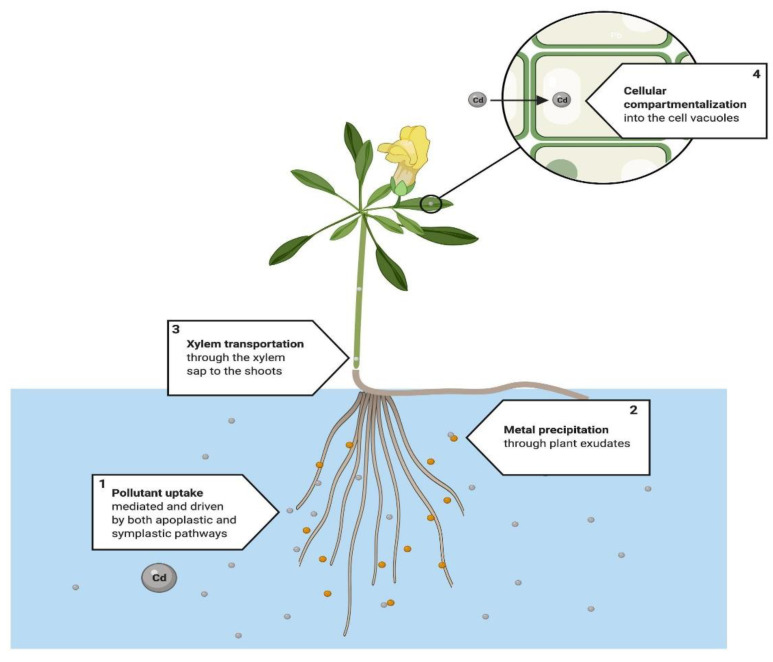
Proposed phytostabilization system. The main actions taken into the plant physiology are: (1) the root uptake of the metal, to start the mobilization into the plant tissues. (2) At the same time, in the areas near the roots start a precipitation process, where some beneficial bacteria may be associated with some metabolites produced by the plant’s root. Finally, (3) the metals are mobilized to the aerial section, and (4) are compartmentalized in different tissues and different organelles, mainly in vacuoles, chloroplasts and sometimes mitochondrias [127,128,129].

This is a process occurring in the rhizosphere (Figure 6) to stabilize and immobilize pollutants from the substrate [126], which is the principal advantage of this technique considering that there is no need for harvesting from the source location [127]. This process is compatible with the sequestration of pollutants, mainly metals, within the rhizosphere [111,112,126].

Some of the studies reviewed showed a relation between the pollutant characteristics and the plant physiology, such as nutrient uptake, chlorophyll concentration, and biomass production, among others [56,58,59,127]. This may be related to what species are used to stabilize HM, such as those referred to in Table 1.

### 4.5. Phytovolatilization

**Figure 7:**
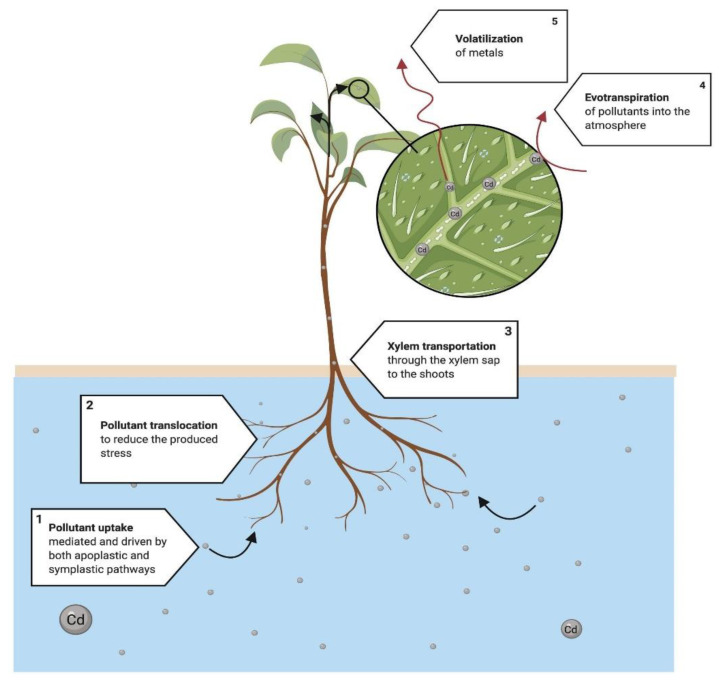
Proposed mechanism of phytovolatilization. (1) The process begins with the uptake and mobilization of the pollutant in the roots. (2) At the same time, the translocation of some ions, mainly metals, starts to reduce the produced stress in plant tissues. (3) The rest of the pollutants are transported to the photosynthetic area and start two main processes: (4) the evapotranspiration acts similarly to a vacuum to extract, in the presence of water, the pollutants and transport them into the atmosphere; meanwhile, (5) the combination of temperature and UV rays volatilizes the pollutants near the stomata of the leaves, mobilizing a great portion of the pollutants into the atmosphere, but transformed into less ecotoxic components/metabolites [130].

Phytovolatilization (Figure 7) is described as removing and fractionating pollutants within plant air spaces and later diffusion into the ambient air [130,131]. Thus, plants may volatilize specific metals, such as selenium [127], and volatile organic compounds (VOCs) [132]. The mechanisms that explain the mobilization of HMs are explained in Figure 7. This figure provides information on why most of the processes of the phytoremediation of HMs are focused on processes other than phytovolatilization; however, some metals, such as Cd, may be volatilized following this process.

### 4.6. Relationship Root-Microorganisms

The relationships involving the root of plants and microorganisms are well described in soil [133]; even if an aquatic medium may modify these interactions, overall, the processes induced in rhizosphere may be similar [110,111]. In Table 2, we show a comprehensive description of the interactions in water phytoremediation where microorganisms participate, mostly plant growth-promoting rhizobacteria (PGPR).

### 4.7. Environmental Characteristics That Influence Water Phytoremediation

**Figure 8:**
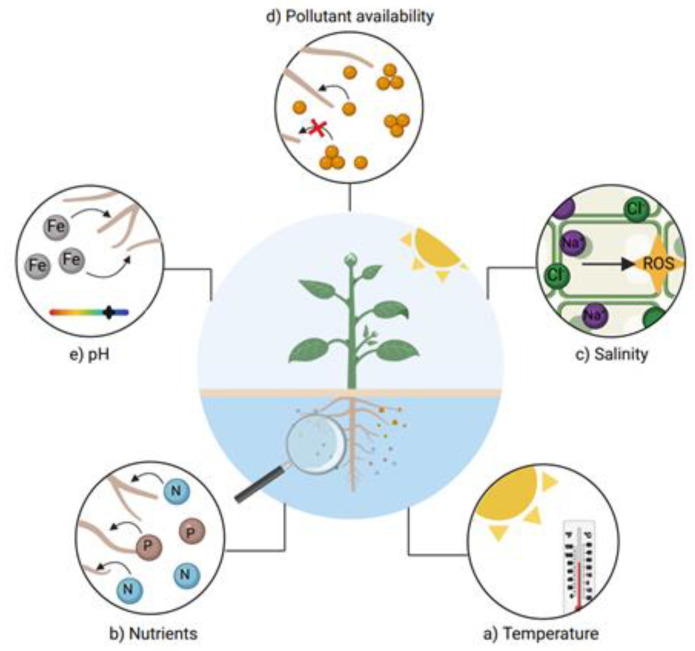
Main factors affecting water phytoremediation. Temperature, nutrients bioavailability, salinity, pollutant (HM) availability and pH are some factors that may delay phytoremediation processes. The interactions occurring in roots play an important role in systems where mobilization of the pollutant via the root is essential, such as HM phytoremediation.

The environmental characteristics of the water should be taken into account when phytoremediation systems are induced. Studies have shown the impact on different values during phytoremediation processes in water (Figure 8). For example, the iron removal capacity in some cases depends on the pH values, due its oxidation–reduction potential [143], and for other HMs such as Zn and Cd, bioavailability is directly related with higher pH values [144].

On the other hand, temperature is an important factor in water phytoremediation systems, since many plant species’ growth may be limited by this factor [145]. Other characteristics, such as salinity, limit the species that may be useful for the removal of HMs in water, since not every plant is halotolerant [146]; furthermore, salinity directly affects ionic homeostasis in cells and promotes the formation of reactive oxygen species (ROS) [147]. The uptake mechanisms involved in the uptake of HMs are directly related to the speed of removal [148]; the higher the bioavailability of the metals, the higher the removal speed. Finally, the nutrients absorbed through roots can accelerate or decrease the uptake of HMs. Healthy plants with the correct nutrient balance can absorb water and nutrients faster [149], thus leading to an increased uptake of HMs.

## 5. Obtained Patents in the Field of Water Phytoremediation

Patents are directly related to applied science. By describing the process and validating it, research becomes reliable and applicable worldwide. Only 9 out of the 40 reviewed species have patents related to the contaminated water treatment (Table 3). Most of the species have patents related to production, agroalimentary systems, herbal extracts, or the control of UFO species. Species such as *Azolla pinnata*, [140,150,151], *A. pinnata*, *P. stratoes* [152], *S. polyrhiza* [153], and *Phragmites australis* [154,155,156] have been registered as phytoremediators of various pollutants, and have many other usages, such as insecticides [157], low-cost supplemental food for aquaculture [158], and medical applications, such as antioxidant, anti-inflammatory, anti-apoptotic [159] and bioactive constituents in the extracts [160,161].

**Figure 9:**
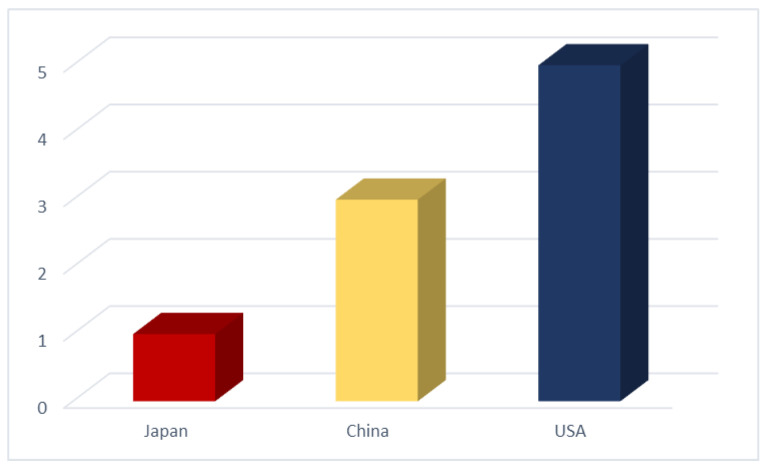
Only 9 patents related to water phytoremediation have been submitted, out of the 40 species that have been reported. In terms of the data collected in Table 2, the two countries with the most patents related with water phytoremediation are USA and China, followed by Japan.

The patents that described the exact participation and phytoremediation processes were only those for *Pteris vittata*, *Eichhornia crassipes*, *Pistia stratiotes*, and *Hydrilla verticillata*, and most of them related to rhizofiltration and phytostabilization. The other patents referred to the processes as “plants” or just “systems” without delving into phytoremediation processes’ mechanisms. There were five patents from the USA, three patents from China, and the last one out of the nine phytoremediation patents was from Japan (Figure 9, Table 3). From Table 1, we can observe that the USA and China have different studies related to phytoremediation in water, with a primary focus on hydroponic systems, to remove some organic and inorganic pollutants. There are nearly 250 reports in the last decade that infer information about phytoremediation capabilities of the reviewed species. However, the patents associated with these species barely cover nine (Figure 9), with four of them belonging to the five most representative families found in this study, which leads to an unexplored area to find and support applications for the other species. However, most of the mechanisms described in the proposed patents are limited in their application, rather than in the diversity of plants that can be used.

## 6. Current Challenges and Literature Gaps

The unexplored field ahead, not only from genera but entire families, is a viable option to follow in current research. From all the species described as phytoremediators, just a few are associated with patents in the phytoremediation field, since water phytoremediation mechanisms have limited application. This is relevant since the data analyzed may produce more knowledge than the total registered, as described in this review, as patents are based on applied research. Another possible reason is the lack of data for each species, not only to classify them as phytoremediators but also to design an ascertainable and replicable system that can be patented. The phytoremediation processes need to be exploited to understand several in-depth variables that directly or indirectly influence bioremediation.

Another key point is selecting one species to construct a phytoremediation system. We need to consider different factors, since many contaminants do not have the same chemical structure and characteristics. This is an essential limiting factor for phytoremediation systems. The uptake mechanisms are, in several cases, affected by the microbiota coexisting near the root of the plants in soil and water systems. One main reason for the low uptake and mobilization of the contaminants is the root cells’ lack of capacity to capture the compound. The last key point to highlight is the limitation of the contaminant uptake, mediated by the water’s physicochemical conditions, such as pH, electric conductivity, and cation exchange capacity, among others. These factors can be modified or regulated to improve and accelerate the ecotoxic compound’s mobilization, which needs to be treated.

The solution to each key point is as vast as the total number of plant species around the world. Every species, and even each individual, has a wide variety of strategies to mobilize certain compounds, including ecotoxic. Suppose one contaminant remains immovable from the substrate or sediments in the water. In that case, the combination of different plant species may help in different ways, from breaking chemical bonds to volatilizing the entire compound or even reducing the bioavailability to other organisms. Describing exact processes in water phytoremediation systems may help to provide new ways to understand phytoremediation. However, there is a dire need to emphasize the importance of exploring species, genera, and entire families’ phytoremediation capabilities. We need to address pollution issues from a sustainable and interdisciplinary perspective. Current data show that the cost–benefit of phytoremediation may be higher than traditional treatments, such as chemical or physical. The infrastructure of many countries can not apply efficient conventional techniques. From this perspective, phytoremediation is a viable strategy to solve many countries’ water issues, meaning that it is an essential and urgent topic for research.

## 7. Conclusions

Water pollution is an issue that needs to be addressed from an ecologically friendly perspective, since many diseases are directly related to polluted drinking water. Phytoremediation opens up opportunities aiding in the removal of contaminants that may be hazardous to many organisms. There are a wide range of strategies and mechanisms described in phytoremediation, and there is more experimental information in soil than water. However, the application of these systems may remain uncertain. On one hand, the related data showed the lack of information associated with many plant species compared with the total species in general. This is an important issue, since many plant species from the same genera showed phytoremediation capabilities.

From the 40 species reviewed in total, the wide variety of phytoremediators grow naturally into or along areas associated with water bodies. This is a necessary trait to consider, since the uptake mechanisms of terrestrial and aquatic plants may differ; however, the literature showed that the association between microorganisms and root may not be limited in water systems. Nevertheless, the overall phytoremediation system can explain the uptake and mobilization dynamics of the contaminant without considering this factor. By comparing the results, we can address many factors that limit the analysis, such as the results presented in different measurement units. Even though the conversion of units can be applied, the methodology described in various studies might add some noise effects, since the reactive conditions, time of observation, and many other variables are not equal. Different species behave differently towards each contaminant. Even if one species shows positive results in removing specific pollutants, it might not be useful for other types of compounds.

The more studies that address the importance of biodiversity in terms of water HM phytoremediation, the more possibilities can be explored to provide and explain precise mechanisms for HM removal in water, using different species of plants to expand the proposed systems and the associated patents.

## Figures and Tables

**Table 1:** Data from 40 species analyzed in studies related to phytoremediation in water. The presented data were published in studies between 2000 and 2020.

Plant Species	Family	Country	Contaminants Treated	Phytoremediation Process	Results	References
*Arundo donax*	Poaceae	Pakistan	As	Phytoextraction	Removal of at least 15% of the pollutant in the treatment of 600 μg L^−1^	[35]
*Azolla caroliniana*	Salvi-niaceae	India	Heavy metals in metal enriched fly ash pond (Cr, Pb, Cu and Ni)	Phytoaccumula-tion	High sequestration of metals (175–538 and 86–753 mg kg^−1^ plant tissue) BCF 1.7–18.6 and 1.8–11.0.	[36]
*Azolla filiculoides*	Salvi-niaceae	Chile, Israel	Cd, Cu, Pb	Phytoextraction	High concentration in plant tissues, more than 1000 micrograms per kg^−1^,	[37,38]
*Azolla pinnata*	Salvi-niaceae	India, Nigeria	Hg, Cd, Zi, Fe	Phytoextraction	Metal content decreased to 70–94%, there is no significant removal of Fe, but Zi decreased more than 30%	[39]
*Canna indica*	Cannaceae	India	F	Phytoaccumula-tion	95% fluoride removal	[40]
*Ceratophyllum demersum*	Ceratophyllaceae	Egypt	Cr, Pb	Phytoaccumula-tion	95% removal of lead and 84% of chromium	[41]
*Cyperus alternifolius*	Cyperaceae	India	F	Phytoaccumula-tion	65% fluoride removal	[40]
*Eichhornia crassipes*	Pontederiaceae	India, Nigeria	As, Hg, Ni, Pb, Zn, Cu, Ag	Phytoaccumula-tion	Acummulation from 26 mg/kg to 327 mg/kg in dry weight	[42,43]
*Eleocharis acicularis*	Cyperaceae	Japan	Cu, Zn, As, Cd, Pb	Phytoextraction	Remotion higher than 90% of the heavy metals	[44]
*Helianthus annuus*	Asteraceae	Pakistan	Ni, Pb	Phytoextraction	More than 50% of removal, 17 mg Kg^−1^ in plant tissue	[45]
*Hydrilla verticillata*	Hydro-charitaceae	India, China	F, As, and other heavy metals	Phytoaccumula-tion, Phyto-degradation	Maximum removal 24.4% at 2.5 ppm without dramatically affecting associated physiological parameters, and the resultant degradation products are non-toxic	[46,47,48]
*Ipomoea aquatica*	Convolvulaceae	Iran, Sri Lanka	Pb, Cr	Rhizofiltration	The highest BCF (4179.07) value was registered in root tissue (0.63 mg L^−1^ Pb) More than 90% Cr(VI) sequestrated in leaves and steams. In none of the Cr(VI) dosing experiments did the I. aquatica show toxicity symptoms.	[49,50]
*Iris pseuda-corus*	Iridaceae	Spain	Cr, Zn	Rhizofiltration	59.97 mg Cr and 25.64 mg Zn in roots	[51]
*Juncus effusus*	Juncaceae	China	Pb	Phytodegradation	Concentrations higher than 2000 mg kg^−1^ in roots	[52]
*Lemna gibba*	Araceae	Germany	U, As	Phytoextraction	Accumulation in plant tissue, around 500 mg kg^−1^	[53]
*Lemna minor*	Araceae	Pakistan, Iran	Heavy metals in contaminated effluents	Phytoaccumula-tion	Considerable reduction in every metal in municipal effluent	[27]
*Lepironia articulata*	Cyperaceae	USA	Pb	Rhizofiltration	More than 500 mg/kg in its plant tissue (roots) and 217 of BCF value	[54]
*Lolium perenne*	Poaceae	France	Cr	Phytostabilization	High accumulation in roots, higuer than 2000 μg^−1^ DW	[55]
*Ludwigia stolonifera*	Onagra-ceae	Egypt	Cd, Ni, Zn, Pb	Phytostabilization	Bioaccumulation and translocation factor showed positive interaction for the uptake of metals highlighted	[56]
*Mentha aquatica*	Lamiaceae	Lebanon	Ni	Rhizofiltration	8327 mg kg^−1^ accumulated mainly in root tissue	[57]
*Myrio-phyllum aquaticum*	Haloragaceae	Italy	Cd, Cr, Ni, Zn	Phytoaccumulation	High accumulation in plant tisssue at high concentrations, more than 500 μg g^−1^ DW	[58]
*Myrio-phyllum triphyllum*	Haloragaceae	Turkey	Cd	Phytoaccumu-lation	17.03 μg Cd accumulation was found in a gram in dried sample	[59]
*Myrio-phyllum elatinoides*	Haloragaceae	China	B	Phytoaccumulation	Maximal tissue accumulation in shoot tissue and root section (1296.5 and 350.7 mg/kg, each one)	[60]
*Nelumbo nucifera*	Nelum-bona-ceae	India	Cd, Co, Cu, Ni, Pb and Zn	Phytoextraction	Accumulation in tissue more than 340 ppm of metals	[61]
*Oenanthe javanica*	Apiaceae	USA	Hg	Phytoaccumulation	More than 1 mg/kg remediated and 807 of BCF value	[62]
*Phragmites australis*	Poaceae	Saudi Arabia, Denmark	Cd, Pb, Ni	Rhizofiltration	High concentration in roots, more than 3 mg kg^−1^	[63]
*Pistia stratiotes*	Araceae	USA, India	Cd, Cu, Fe, Hg	Phytoextraction and rhizofiltration	Accumulation of Cd in roots (more than 10 mg kg^−1^), Cu, Fe and Hg concentrations from 1 to 15 mg kg^−1^ DW.	[64,65]
*Plantago major*	Plantaginaceae	Switzerland	Pb	Rhizofiltration	High uptake, more than 20 mg/kg of Pb in root tissue	[66]
*Potamo-geton natans*	Potamogetonaceae	Sweden	Zn, Cu, Cd, Pb	Rhizofiltration	Highest accumulation found in the roots	[67]
*Pteris vittata*	Pteridaceae	USA	As	Phytoaccumulation	Reduced arsenic concentration by 98.6%	[68]
*Salvinia biloba*	Salviniaceae	Brazil	Pb	Phytoextraction	Almost 90% of Pb remotion	[69]
*Salvinia minima*	Salviniaceae	Mexico	Pb, As	Phytoaccumu-lation	More than 34 mg/g Pb in dry weight tissue and high As uptake, with 0.5 mg/g DW).	[70]
*Salvinia molesta*	Salviniaceae	Brazil	As	Phytoaccumu-lation	Accumulation in leaves, highest accumulation 148.63 μg g^−1^ DW	[71]
*Salvinia natans*	Salviniaceae	India	Zn, Cu, Ni, Cr	Phytoaccumu-lation	High removal, more than 50% average for each metal	[72]
*Spirodela polyrhiza*	Araceae	Japan	As	Phytoaccumu-lation	Accumulations on DW tissue higher than 0.35 μmol/g for arsenate and around 7.6 nmol/g DW for DMAA	[73]
*Trapa natans*	Lythraceae	India	Heavy metals in wastewater	Phytoaccumu-lation	Metal contents translocated in leaves, whereas most contents of Cr and Pb were accumulated in the root.	[74]
*Typha domin-gensis*	Typhaceae	Egypt, Brazil	P, Na, K, Zn, Hg	Phytoextraction	Reduced P, Na, K almost in 80%, reduced Zn in 10% with respect to initial values, Reduces 99.6 ± 0.4% of the mercury in contaminated water	[75,76]
*Typha latifolia*	Typhaceae	Italy	Cu, Zn	Phytoextraction	Higher accumulation of Zinc, more than 55 mg Kg DW in root tissue	[77]
*Vallisneria natans*	Hydrocharitaceae	China	As	Rhizo-filtration	High accumulation in roots (more than 200 mg/kg^−1^ DW of As (IV))	[78]
*Wolffia globosa*	Araceae	China, Thailand	As, Cd, Cr	Phyto-accumu-lation	Accumulate more than 1000 mg As kg^−1^ in DW tissue, Max accumulation Cd 5931 µg/g DW. 3500 µg/g DW Cr	[79,80]

**Table 2:** Different processes in the root-microorganism association during phytoremediation of HMs in water.

Microorganism	Process	Reference
PGPR (*Paenibacillus mucilaginosus, Sinorhizobium meliloti*)	Increase the bioavailability of metals	[134]
PGPR (*Pseudomonas* spp.)	Increase water uptake in roots, increasing HM mobilization	[135]
PGPR (*Stenotrophomonas maltophilia*)	Reduce toxicity of HMs, increasing bioaccumulation factor (BF)	[136]
PGPR (non specified)	Transformation of HMs into less toxic compounds for faster uptake	[137]
PGPR (*Planomicrobium chinense, Bacillus cereus*)	Increase biomass gain and root growth during HM stress	[138]
PGPR (*Bacillus* spp.)	Reduction in oxidative stress, increasing metabolite production	[139]
*Chryseobacterium* sp.	Creation of antagonistic metabolites to improve resistance to pathogens	[140]
PGPR (*Pseudomonas fluorescence, Bacillus subtilis*)	Increase HM uptake, especially Pb and Ni	[141]

PGPR have shown positive interactions with plant roots during physiological stress, from inducing metabolite production to enhancing biomass production [142], and even the way in which nutrients are recycled has similar mechanisms in water and soil [110], although some processes, such as the fate of metabolites, can vary between terrestrial and aquatic systems [111]. Nevertheless, dynamics on water may express different interactions and may be studied in future research.

**Table 3:** Nine species associated with the patents in phytoremediation processes or monitoring.

Plant Species	Patent	Patent Number	Reference
*Azolla pinnata*	Water purification system	EP0333218B1	[162]
*Spirodela polyrhiza*	Purification method of wastewater	WO2012029736A1	[163]
*Eichhornia crassipes*	Purifying algae-type eutrophic contaminated water bodies at a source	CN102524084A	[164]
*Hydrilla verticillata*	The invention discloses a method for removing nitrogen and phosphorus in a water body	CN102311173A	[165]
*Iris pseudacorus*	Waste-water purification plant	US7718062B2	[166]
*Myriophyllum triphyllum*	Marine biomass reactor	WO2018140449A1	[167]
*Phragmites australis*	Waste treatment systems, biological restoration of water body, system and method for removal of pollutants from water	US7361268B2	[168]
*Potamogeton natans*	Method for repairing water ecology, purifying method, waste treatment process	US6652743B2	[169]
*Pteris vittata*	Method for removing arsenic from soil and water	CN105945042A	[170]

## Data Availability

All the references included in this review has their corresponding citation for further discussion and analysis.
